# Chromosome-scale genomes of commercially important mahoganies, *Swietenia macrophylla* and *Khaya senegalensis*

**DOI:** 10.1038/s41597-023-02707-w

**Published:** 2023-11-25

**Authors:** Sunil Kumar Sahu, Min Liu, Guanlong Wang, Yewen Chen, Ruirui Li, Dongming Fang, Durgesh Nandini Sahu, Weixue Mu, Jinpu Wei, Jie Liu, Yuxian Zhao, Shouzhou Zhang, Michael Lisby, Xin Liu, Xun Xu, Laigeng Li, Sibo Wang, Huan Liu, Chengzhong He

**Affiliations:** 1 State Key Laboratory of Agricultural Genomics, Key Laboratory of Genomics, Ministry of Agriculture, BGI Research, Shenzhen, 518083 China; 2 BGI Life Science Joint Research Center, Northeast Forestry University, Harbin, 150400 China; 3 College of Science, South China Agricultural University, Guangzhou, 510642 China; 4 College of Life Sciences, Chongqing Normal University, Chongqing, 400047 China; 5 Forestry Bureau of Ruili, Yunnan Dehong, Ruili, 678600 China; 6 State Key Laboratory of Tree Genetics and Breeding, Research Institute of Forestry, Chinese Academy of Forestry, Beijing, 100091 China; 7 Laboratory of Southern Subtropical Plant Diversity, Fairy Lake Botanical Garden, Shenzhen, Chinese Academy of Sciences, Shenzhen, 518004 China; 8 Department of Biology, University of Copenhagen, Copenhagen, DK-2100 Denmark; 9 Guangdong Provincial Key Laboratory of Genome Read and Write, BGI-Shenzhen, Shenzhen, 518083 China; 10 National Key Laboratory of Plant Molecular Genetics and CAS Center for Excellence in Molecular Plant Sciences, Institute of Plant Physiology and Ecology, Chinese Academy of Sciences, Shanghai, 200032 China; 11 Key Laboratory for Forest Genetic & Tree Improvement and Propagation in Universities of Yunnan Province, Southwest Forestry University, Kunming, 650224 China

**Keywords:** Comparative genomics, Next-generation sequencing

## Abstract

Mahogany species (family Meliaceae) are highly valued for their aesthetic and durable wood. Despite their economic and ecological importance, genomic resources for mahogany species are limited, hindering genetic improvement and conservation efforts. Here we perform chromosome-scale genome assemblies of two commercially important mahogany species: *Swietenia macrophylla* and *Khaya senegalensis*. By combining 10X sequencing and Hi-C data, we assemble high-quality genomes of 274.49 Mb (*S. macrophylla*) and 406.50 Mb (*K. senegalensis*), with scaffold N50 lengths of 8.51 Mb and 7.85 Mb, respectively. A total of 99.38% and 98.05% of the assembled sequences are anchored to 28 pseudo-chromosomes in *S. macrophylla* and *K. senegalensis*, respectively. We predict 34,129 and 31,908 protein-coding genes in *S. macrophylla* and *K. senegalensis*, respectively, of which 97.44% and 98.49% are functionally annotated. The chromosome-scale genome assemblies of these mahogany species could serve as a vital genetic resource, especially in understanding the properties of non-model woody plants. These high-quality genomes could support the development of molecular markers for breeding programs, conservation efforts, and the sustainable management of these valuable forest resources.

## Background & Summary

The stability of forest ecosystems is increasingly being threatened by factors such as global climate change and unrestricted anthropogenic exploitation^1^. Therefore, for the conservation and development of timber species, it is important to generate genomic information and decode the underlying genetic architecture and regulatory mechanisms to improve forest productivity, adaptation, resilience, and sustainability^2,3^. In recent years, scientists have made significant progress in sequencing and analyzing the genomes of timber tree species like *Populus trichocarpa*^4^, *Eucalyptus grandis*^5^, *Tectona grandis*^6^, *Dalbergia sissoo*^7^, and *Hopea hainanensis*^3^, which has provided valuable insights into the genetic basis of traits such as wood formation, growth, and adaptation to environmental stress^2^. Genomics-based approaches can be used to directly and significantly improve the productivity and adaptability of timber species. These approaches can be used to modify one or more genes in the genomes of timber species, or to identify effective genetic markers and genes for molecular breeding. Genomic research can also accelerate the generation of knowledge in systems biology, which is important for the development of computational genomics^8^. Computational genomics has opened up new ways of identifying genes that regulate complex traits, and through gene stacking and genome editing, customized timber species with special applications can be designed^9^. Forest trees are essential for maintaining biodiversity in terrestrial ecosystems and for producing fiber, fuel, and biomass^10^. Therefore, the importance and legitimacy of forestry studies, including genomics, will be a higher priority in the future.

Mahogany is a tropical hardwood known for its durability, stability, and beautiful reddish-brown color of its wood, and is commonly used in the manufacturing of fine furniture, cabinetry, flooring, and musical instruments^11^. *Swietenia macrophylla*, commonly known as large-leaf mahogany, is a tropical timber species in the Meliaceae family that can tolerate a wide range of soils and environmental conditions. It can grow up to 40 meters tall, have a diameter of up to two meters, and live for several centuries^12^. *S. macrophylla* is one of three species that produces genuine mahogany timber (Swietenia) and is famous for its high-quality wood, which plays an important role in the international mahogany market. The wood is used principally for making furniture, musical instruments, interior fittings and ship building^13^. Furthermore, *S. macrophylla* contains a variety of bioactive compounds such as phenols, flavonoids, terpenoids, and alkaloids, which are rich in medicinal value^14,15^. Overall, the study of *S. macrophylla* highlights the urgent need to protect this valuable and threatened species. Through better management practices, forest conservation, and the sustainable use of this resource, we can ensure the long-term survival of *S. macrophylla* and other important tropical hardwood species.

*Khaya senegalensis* is another important species of deciduous tree in the Meliaceae family that is native to Africa. The wood *K. senegalensis* is prized for its beauty and durability, and it is used for a variety of purposes, including carpentry, interior trim, and construction. Traditionally, the wood was also used to make dugout canoes, household implements, djembe drums, and fuel wood^16,17^. It is also used in traditional African folk medicine, and has been shown to be effective in treating a variety of ailments, including malaria, fever, and diarrhea. Overall, *K. senegalensis* is an important tree with a variety of uses. It is a valuable source of timber, and it has the potential to be used in a variety of medical applications. To date, genome sequences of several important tree species of the Meliacea family have been sequenced such as *Toona sinensis*^18^, *Toona ciliata*^19^, *Azadirachta indica*^20^, *Xylocarpus rumphii*, *X. moluccensis* and *X. granatum*^21^.

Here, we construct high-quality genomes of *S. macrophylla* and *K. senegalensis* using a combination of 10x reads and Hi-C sequencing data. We predict 34,129 (*S. macrophylla*) and 31,908 (*K. senegalensis*) protein-coding genes. We also identify 187 and 123 miRNAs, 648 and 844 tRNAs, 249 and 186 rRNAs from the *S. macrophylla* and *K. senegalensis* genomes. Although the draft genome of *S. macrophylla*^21^ has been published previously, it lacks Hi-C data, and our study elevates the genome to the chromosome-scale with a longer N50 by combining Hi-C data, resulting in a higher-quality genome assembly.

## Methods

### Sample collection, library construction and sequencing, genome size evaluation

The fresh young leaves of *Swietenia macrophylla* (HCNGB_00002344) and *Khaya senegalensis* (HCNGB_00002341) were collected from Ruili, Yunnan, China (24°03′04.4″N 97°56′16.9″E), and stored in the Herbarium of China National GeneBank (HCNGB) (Supplemental Figs. 1–2). DNA was extracted using CTAB (cetyltrimethylammonium bromide)^22^, then GEM and barcode sequences were generated based on the standard protocol (Chromium Genome Chip Kit v1, 10X Genomics, Pleasanton, USA) for *S. macrophylla* and *K. senegalensis*. The barcode libraries were followed by sequencing on the BGISEQ-500 platform to generate 150 bp read pairs^23^. Finally, we generated 1283.02 million reads and 192.45 Gb of raw data in *S. macrophylla* while *K. senegalensis* has 1141.22 million reads and 171. 18 Gb of raw data (Supplemental Table S1).

We also collected fresh young leaves, and branch samples from each species to collect xylem and phloem tissues, and RNA was extracted using the PureLink RNA Mini Kit (Thermo Fisher Scientific, Carlsbad, CA, USA) following the standard protocol to construct RNA libraries using the TruSeq RNA Sample Preparation Kit manual (Illumina, San Diego, CA, USA). RNA libraries were then sequenced on the BGISEQ-500 platform (paired-end, 100-bp reads or 150-bp reads) and the RNA reads were filtered to generate 241.63 million clean reads and 45.88 Gb of clean data for *S. macrophylla* as well as 517.49 million clean reads and 104.53 Gb of clean data for *K. senegalensis* (Supplemental Table S2) by the Trimmomatic^24^ with the parameters:ILLUMINACLIP:adapter.fa:2:30:20:8:true HEADCROP:5 LEADING:3 TRAILING:3 SLIDINGWINDOW:5:8 MINLEN:50.

For Hi-C libraries, MboI restriction enzymes were used and constructed according to the *in situ* ligation protocol^25^. The MboI-digested chromatin was end-labelled with biotin-14-dATP (Thermo Fisher Scientific, Waltham, MA, USA) and used for *in situ* DNA ligation. The DNA was extracted, purified, and then sheared using Covaris S2 (Covaris, Woburn, MA, USA). The DNA libraries were sequenced on a BGISEQ-500 after A-tailing, pull-down and adapter ligation to produce 100-bp read pairs which generated 1483.63 million reads and 148.36 Gb of Hi-C raw data for *S. macrophylla* and 1519.79 million reads and 151.98 Gb of Hi-C raw data for *K. senegalensis* (Supplemental Table S1).

A k-mer (k = 21) analysis was constructed using the obtained DNA sequencing reads from the 10X libraries which were filtered using SOAPnuke^26^ with the parameters (-l 10 -q 0. 1 -n 0. 01 -Q 2 -d–misMatch 1–matchRatio 0.4) to estimate genome sizes, proportion of repeat sequence and heterozygosity. The k-mer frequency distribution analysis was performed using the following formula:

$$Gen=Num\ast \left(Len-17+1\right)/K\_Dep$$

**Fig. 1:**
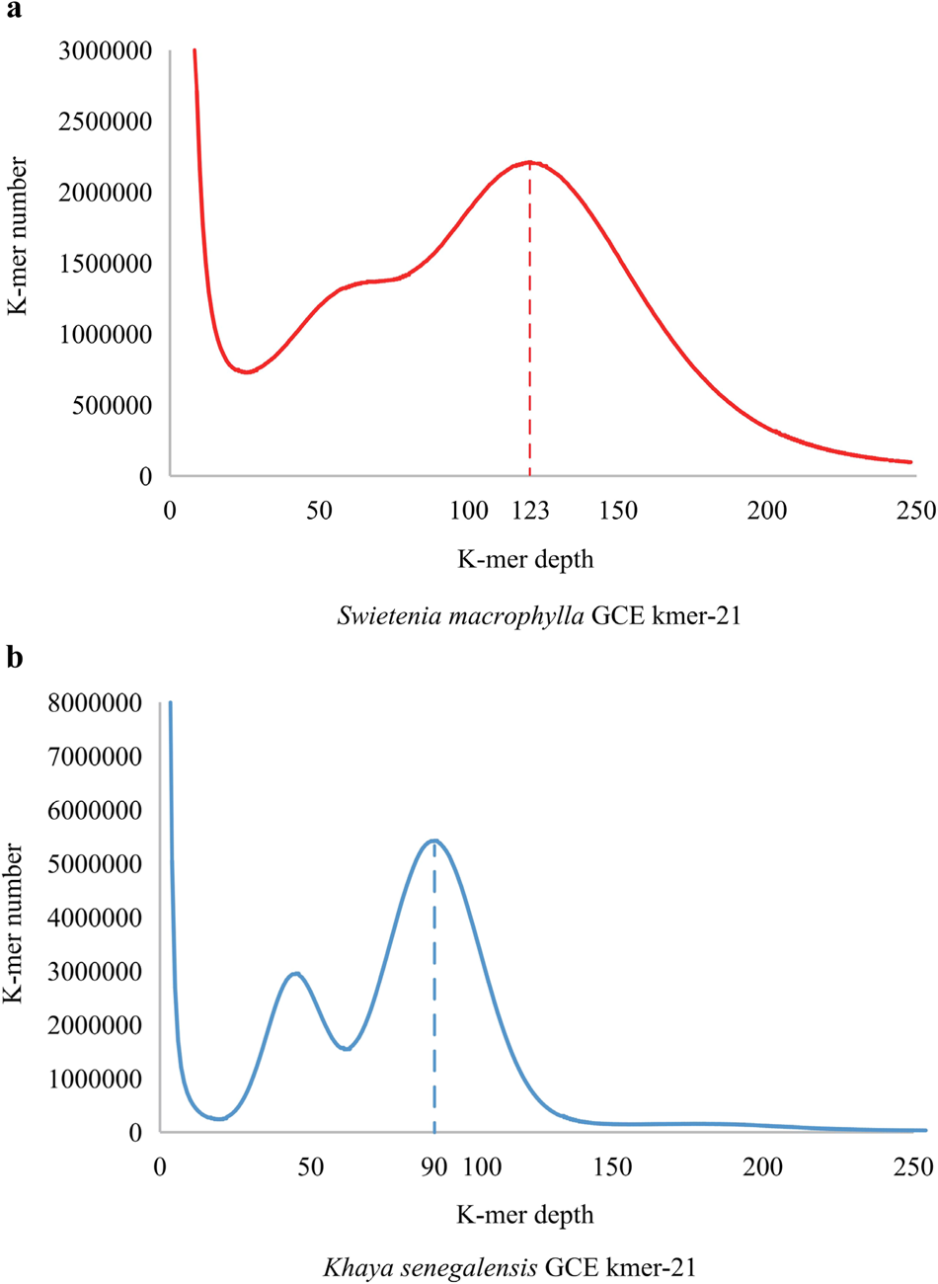
21-kmer distribution in two mahogany genomes. (**a**) *S. macrophylla*. (**b**) *K. senegalensis*. The dashed line indicates the expected K-mer depth.

Where *Num* represents the read number of reads used. *Len* represents the read length, *K* represents the k-mer length, and *K_Dep* refers to where the main peak is located in the distribution. The distribution of 21-kmers showed that the heterozygosity and duplication rate of the genome were respectively 1.00% and 20.14% in *S. macrophylla*, 0.73% and 42.60% in *K. senegalensis*, with genome sizes of 274.49 Mb (*S. macrophylla*) and 406.50 Mb (*K. senegalensis*) (Fig. 1 and Supplemental Table S3).

### Genome assembly, evaluation, and repeat annotation

**Fig. 2:**
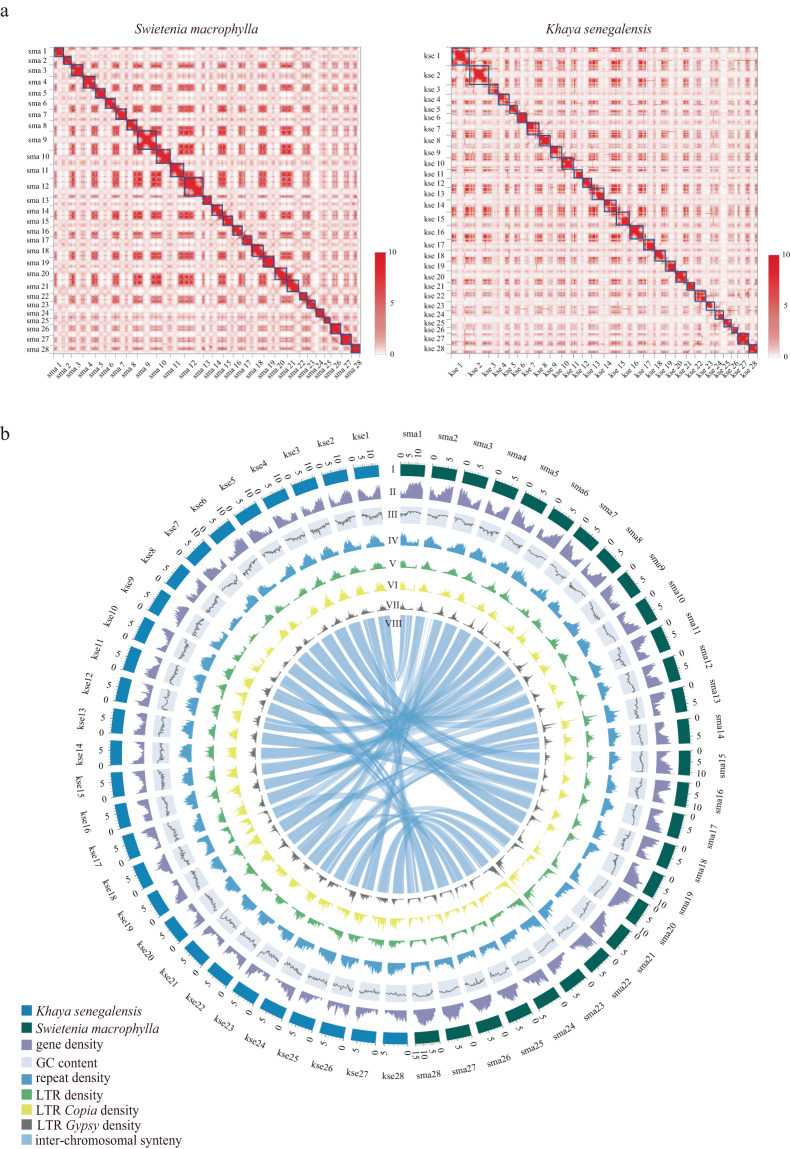
Hi-C and Circos plots of two mahogany genomes (**a**) Hi-C map of the *S. macrophylla* and *K. senegalensis* genome showing genome-wide all-by-all interactions. The map shows a high resolution of individual chromosomes that are scaffolded and assembled independently. The heat map colors ranging from light pink to dark red indicate the frequency of Hi-C interaction links from low to high (0–10). (**b**) Circos plot of *S. macrophylla* and *K. senegalensis* genome. Concentric circles from outermost to innermost show (I) chromosomes and megabase values, (II) gene density, (III) GC content, (IV) repeat density, (V) LTR density, (VI) LTR *Copia* density, (VII) LTR *Gypsy* density and (VIII) inter-chromosomal synteny (features II-VII are calculated in non-overlapping 200 Kb sliding windows).

To perform the genome assembly, a *de novo* assembly program Supernova designed to assemble diploid germline genomes using Linked-Reads (10X library sequences) was used with the default parameters and exported into fasta format using the ‘pseudohap2’ style thereby performing GapCloser^27^ with the parameters “-l 150” to fill the gap. The Hi-C reads were quality controlled and mapped to the genome assembly of each species using Juicer^28^ with default parameters. Subsequently, a candidate superscaffold-level assembly was automatically generated using the 3D-DNA pipeline with default parameters^29^ to correct misjoins, order, orient, and organize scaffolds from the draft assembly. The draft assembly was checked and refined manually in the Juicebox Assembly Tools^30^ (Fig. 2a). The transcriptome sequences were assembled using Bridger tool^31^ and then mapped to the scaffold assembly using BLAT software^32^. The 10X clean reads were preliminarily assembled into scaffold sequences of 290.21 Mb for *S. macrophylla* with 5.76 Mb of Scaffold N50 and 406.50 Mb for *K. senegalensis* with 2.53 Mb of Scaffold N50. The scaffold sequences of two mahogany species were both further anchored onto 28 pseudochromosomes, accounting for 99.38% and 98.05% of the assembled genome. The final chromosome-scale genome assembly was 288.41 Mb with a scaffold N50 of 8.51 Mb in *S. macrophylla* and 370.38 Mb with a scaffold N50 of 7.85 Mb in *K. senegalensis* (Table 1, Supplemental Tables S4-5).

**Table 1:** Genome assembly and assessment statistics.

	*Swietenia macrophylla*	*Khaya senegalensis*
**Genome assembly and annotation**		
Estimated genome size (Mb)	274.49	406.50
Assembly size (Mb)	290.21	377.76
GC content (%)	31.35	28.78
Contig N50 (Kb)	110.84	45.66
Scaffold N50 (Kb)	5758.78	2533.46
Percentage of N content (%)	5.96	12.19
BUSCO completeness of assembly (%)	97.0	96.2
Complete single copy (%)	78.5	74.3
Complete duplicated (%)	18.5	21.9
Total number of genes	34,129	31,908
Average gene length (bp)	3,052.92	3,120.97
DNA mapped reads (%)	97.43	97.68
BUSCO completeness of annotation (%)	93.40	92.20
**Pseudochromosome level assembly**		
Total length of pseudochromosome assembly (Mb)	288.41	370.38
Pseudochromosome number	28	28
Scaffold N50 (Kb)	8510.88	7854.76
Percentage of N content (%)	5.13	10.33
BUSCO completeness of pseudochromosome assembly (%)	95.8	91.6
The rate of pseudochromosome anchored genome (%)	99.38	98.05

Repeating elements were identified using a combination of homology-based and *de novo* approaches using default parameters. For homology-based approaches, we aligned the genome assembly with a known repeat database Repbase v. 21.01^33^ using RepeatMasker v. 4.0.6^34^ for homology-based repeat element characterization. RepeatModeler v.1.0.8^35^ and LTR Finder v. 1.0.6^36^ were used to construct a new repeat library using genome assembly, RepeatMasker v.4.0.6^37^ was followed, used to identify and annotate repeat elements in the genome, and finally TRF v.4. 07^38^ was used to tandem repeats in genomes for annotation (Table 2). We identified 85.08 Mb (29.50%) of repetitive sequences in the *S. macrophylla* genome and 80.85 Mb (21.83%) in the *K. senegalensis* genome. Most of these repeat sequences are Class I (53.57%) retro transposons, including *Copia*, *Gypsy*, LINE and SINE, accounted for 9.04%, 4.87%, 0.54%, 0.03% in *S. macrophylla* and 6.24%, 5.19%, 0.48%, 0.08% in *K. senegalensis* of the entire genome, respectively (Table 2, Supplemental Table S6).

**Table 2:** Genome annotation statistics.

	*Swietenia macrophylla*	*Khaya senegalensis*
Number of protein-coding genes	34,129	31,908
Percentage of functional annotation genes (%)	97.44	98.49
BUSCOs completeness of annotation (%)	93.40	92.20
Average gene length (bp)	3052.92	3120.97
Average exon length (bp)	215.60	229.99
Average exon number per gene	5.58	5.36
Average intron length (bp)	402.79	432.16
Number of miRNAs	187	123
Number of tRNAs	648	844
Number of rRNAs	249	186
Percentage of repeat sequence (%)	29.50	21.83
SINE (%)	0.03	0.08
LINE (%)	0.54	0.48
Copia (%)	9.04	6.24
Gypsy (%)	4.87	5.19

### Gene annotation, functional annotation and noncoding RNAs annotation

The MAKER-P pipeline (version 2.31)^39^ was used to predict protein-coding gene structures based on RNA, homologous protein and *de novo* prediction evidence. Clean transcriptome reads were assembled into inchworms using Trinity (version 2.0.6)^40^ and therefore submitted to MAKER-P as expressed sequence tags for RNA evidence. Protein sequences from the model plant or related species (Supplemental Table S7) were downloaded for two mahogany species and utilized as protein evidence for homology comparisons. In order to perform *de novo* prediction, multiple training sets were created for various *ab initio* gene predictors. The generation of a set of transcripts was initially performed by applying the genome-guided approach of Trinity^40^. Using PASA (version 2.0.2)^41^, these transcripts were then traced back to the genome, creating a collection of gene models with real gene features. For Augustus^42^ training, complete gene models were chosen. Genemark-ES (version 4.21)^43^ was self-trained with default parameters. Based on the aforementioned data, the first round of MAKER-P was run with all default parameters set to “1,” except for “est2genome” and “protein2genome”, which only produced RNA and protein-supported gene models, respectively. The gene models were then used for the training of SNAP^44^. The second and final rounds of MAKER-P were executed using the default parameters to generate the final gene model. The integration of protein-coding genes from *S. macrophylla* and *K. senegalensis* was successfully achieved, resulting in a total of 34129 and 32914 genes, respectively. The average gene length for *S. macrophylla* was determined to be 3052.92 bp, while for *K. senegalensis* it was 3068.00 bp. Additionally, the average lengths of exons and introns were calculated to be 215.60 bp and 402.79 bp, respectively, for *S. macrophylla*, and 230.06 bp and 431.15 bp, respectively, for *K. senegalensis* (Table 2, Supplemental Table S8).

**Fig. 3:**
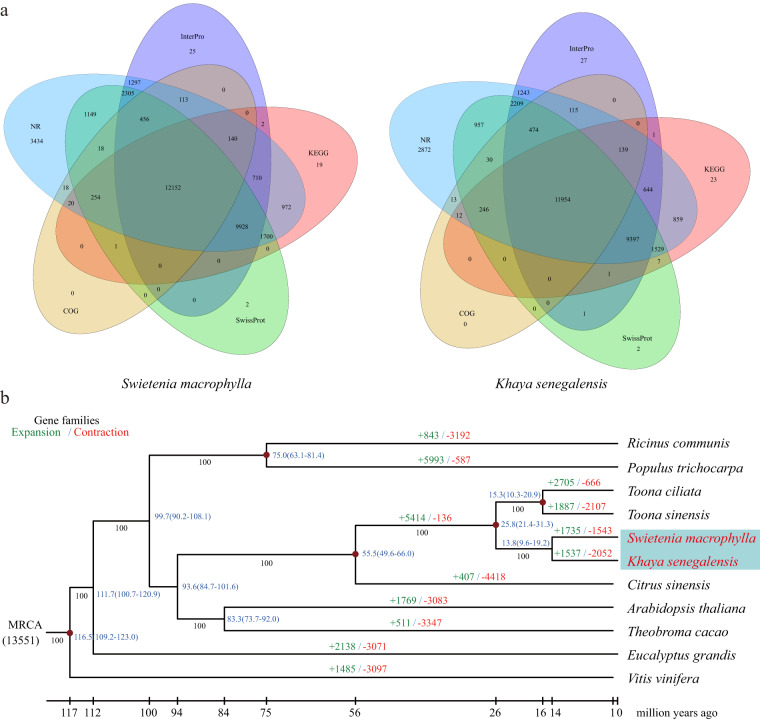
Venn diagram and Phylogenetic position of *S. macrophylla* and *K. senegalensis*. (**a**) Venn diagram of *S. macrophylla* and *K. senegalensi*s. (**b**) The phylogenetic tree constructed by IQtree with ‘-b 100’ using 317 single copy orthologues of two mahogany species and nine other representative plant species. The red nodes indicate fossil calibration nodes. Node labels represent node ages (Mya). The number of expanded gene families (+; green) and the number of contracted gene families (–; red) are shown in each branch. The numbers below the middle of each branch represent the bootstrap values.

Functional annotation of protein-coding genes was performed through the utilization of sequence similarity and domain conservation. This involved comparing the predicted amino acid sequences against publicly available databases. The initial step involved the identification of protein-coding genes by searching for optimal matches against protein sequence databases including the Kyoto Encyclopaedia of Genes and Genomes (KEGG)^45^, the National Centre for Biotechnology Information (NCBI), non-redundant (NR) and COG databases^46^, SwissProt^47^, and TrEMBL. This search was performed using BLASTP with a specified E-value cut-off of 1e-5. Subsequently, InterProScan 55.0 was employed to detect and classify domains and motifs using the Pfam^48^, SMART^49^, PANTHER^50^, PRINTS^51^, and ProDom^52^ databases. Consequently, the annotation rates for *S. macrophylla* and *K. senegalensis* were found to be 97% and 98% respectively (Table 2, Supplemental Table S9). Additionally, a combined total of 12,152 genes (equivalent to 35.61% of *S. macrophylla*) and 11,954 genes (equivalent to 37.46% of *K. senegalensis*) were jointly annotated in five functional databases (Fig. 3a).

To annotate non-coding RNAs, the ribosomal RNA (rRNA) genes were queried against the *A. thaliana* rRNA database using BLASTN V. 2.2.26^53^ with parameter (-e 1e-5 -v 10000 -b 10000). The Rfam database^54^ was queried for microRNAs (miRNA) and small nuclear RNA (snRNA) (tRNAscan-SE^55^ was also employed to scan tRNA). In this study, we successfully isolated ribosomal RNA (rRNA), microRNA (miRNA), and transfer RNA (tRNA) from *S. macrophylla* and *K. senegalensis*. The quantities obtained for *S. macrophylla* were 249 for rRNA, 187 for miRNA, and 648 for tRNA, while for *K. senegalensis*, the quantities were 630 for rRNA, 189 for miRNA, and 844 for tRNA (Table 2, Supplemental Table S10).

### Genome collinearity and Circos plot construction

MCScanX^1^ was used to identify genomic collinearity between the two mahogany species and to obtain their pairs of colinear genes. The file of genomic collinearity generated by MCScanX was combined with the previous genome assembly and annotation results files to construct a circos plot (Fig. 2b). Here, we found that the genomes of two mahogany species share many similar structural features, including: (1) both consist of 28 chromosomes; (2) gene density and GC content show a positive correlation; (3) LTR density is negatively correlated with gene density and GC content; (4) the chromosomes of the two mahogany species show a high degree of collinearity between them, which also supports the close affinity between the two mahogany species. To show the taxonomic position of the sequenced species, the phylogenetic tree was subsequently constructed based on 317 single copy orthologues obtained from OrthoFinder v. 2.3.1^56^ clustering (Fig. 3b). First, MAFFT v. 7.310^57^ was used to conduct multiple sequence alignment for single-copy orthologs protein sequences, and the alignment results were input into IQtree v. 1.6.1^58^ with the parameters “-b 100” to construct phylogenetic tree. The tree building results were rooted and visualized using FigTree v. 1.4 (http://tree.bio.ed.ac.uk/software/figtree). Second, species divergence time was estimated by combining the MCMCTREE module of PAML v. 4.5^59^ and the TToL5 web portal^60^. Finally, we used CAFÉ v. 4.2.1^61^ to analyze the expansion and contraction events of single-copy orthologs. The *S. macrophylla* and *K. senegalensis* diverged ~13.8 Mya and were closest to the genus *Citrus*, which was consistent with *T. sinensis*^18^ and *T. ciliate*^19^ of the same genus. The divergent time between *T. sinensis* and *T. ciliate* was ~15.3 Mya, which overlapped with the results of Wang *et al*.^19^ In addition, these two mahogany species diverged with *A. thaliana* ~93.6 Mya and *P. trichocarpa* ~99.7 Mya, which was similar to He *et al*.^21^ A total of 1735 and 1543 gene families had expanded and contracted in the *S. macrophylla* genome, while 1537 and 2052 gene families had expanded and contracted in the *K. senegalensis* genome, respectively.

## Data Records

All the genomic sequencing raw data were deposited in the Genome Sequence Archive in National Genomics Data Center (NGDC) Genome Sequence Archive (GSA) database with the accession number CRA011793^62^ under the BioProject accession number PRJCA018269^63^. The assembled scaffolds genomes were submitted to the Genome Warehouse under the accession number GWHDONZ00000000^64^, GWHDOOA00000000^65^ of *S. macrophylla* and *K. senegalensis*, respectively. The Chromosome-scale genome assemblies were also submitted to the NCBI under the accession number GCA_032401905.1^66^, GCA_032402905.1^67^ of *S. macrophylla* and *K. senegalensis*, respectively. The raw sequencing data and assembled genomes of *S. macrophylla* and *K. senegalensis* that support the findings of this study have also been deposited into CNGB Sequence Archive (CNSA)^68^ of China National GeneBank DataBase (CNGBdb)^69^ with accession number CNP0004053 and CNP0004052, respectively. The gene annotations, pseudogene predictions, and ncRNA files are available in the Figshare^70^.

## Technical Validation

### Genome assembly and validation of gene prediction

In order to evaluate the quality of genome assembly, we used bwa (version: 0.7.12; mode: aln)^71^ to align the Illumina short reads with the chromosome-level genomes, 97.43% and 97.68% of the Illumina short reads were mapped to the *S. macrophylla* and *K. senegalensis* genomes, respectively (Supplemental Table S11). BUSCO (version 3.0.1)^72^ was used to assess the integrity of our genome assembly, with results showing 97% (*S. macrophylla*), 96.2% (*K. senegalensis*) for scaffold-scale genomes in addition to 95.8% (*S. macrophylla*), 91.6% (*K. senegalensis*) for Chromosome-scale genomes. To assess the results of Hi-C assembly, as shown in the chromosomal interaction heatmap, the intensity of diagonal interactions within each group is higher than the intensity of non-diagonal interactions (Fig. 2a), which was consistent with the principle of Hi-C assisted genome assembly and demonstrated that the genome assembly was accurate. Taken together, the results showed that the genomes of the two mahogany species assembled in this study had a high degree of integrity.

For gene prediction, we used BUSCO (version 3.0.1) to assess the number and proportion of annotated genes from two mahogany species occupying the database of the core set of angiosperm genes (embryophyta_odb10). The results showed that *S. macrophylla* had 1284 genes matched back to the core gene set (93.4%), while *K. senegalensis* had 1268 genes (92.2%), indicating that the annotated gene sets of both mahogany species are highly complete.

### Supplementary information

Supplementary Information

## Data Availability

All software used in this work is in the public domain and their parameters are described in the Methods section. If a software did not mention parameters, the default parameters suggested by the developer were used.
