# Supplementary material for: Chromosome-scale genomes of commercially important mahoganies, Swietenia macrophylla and Khaya senegalensis
Source: Sci Data. 2023 Nov 25;10:832. doi: 10.1038/s41597-023-02707-w (PMC10676371; doi:10.1038/s41597-023-02707-w)
Supplement: Supplementary file 1 — Supplementary Information [file 41597_2023_2707_MOESM1_ESM.pdf]

Content

Supplemental Figure 1 Morphological features of *Swietenia macrophylla* 2

Supplemental Figure 2 Morphological features of *Khaya senegalensis* 3

Table S1 DNA Sequencing statistics of two species 4

Table S2 RNA sequencing statistics of two species 5

Table S3 k-mer analysis of *Khaya senegalensis*, *Swietenia macrophylla* 6

Table S4 The genome assemly results of *Khaya senegalensis*, *Swietenia macrophylla* 7

Table S5 Busco assessment of genome assembly of scaffold 8

Table S6 The repaeat annotation 9

Table S7 Model plant or related species in gene annotation 10

Table S8 The result and busco assessment of gene annotation 11

Table S9 The functional annotation 12

Table S10 The Annotation of non-coding RNA genes in the genomes of *Swietenia macrophylla, Khaya senegalensis* 13

Table S11 DNA reads mapped to the genome 14

Supplementary figures

Chromosome-scale genomes of two commercially important mahogany species

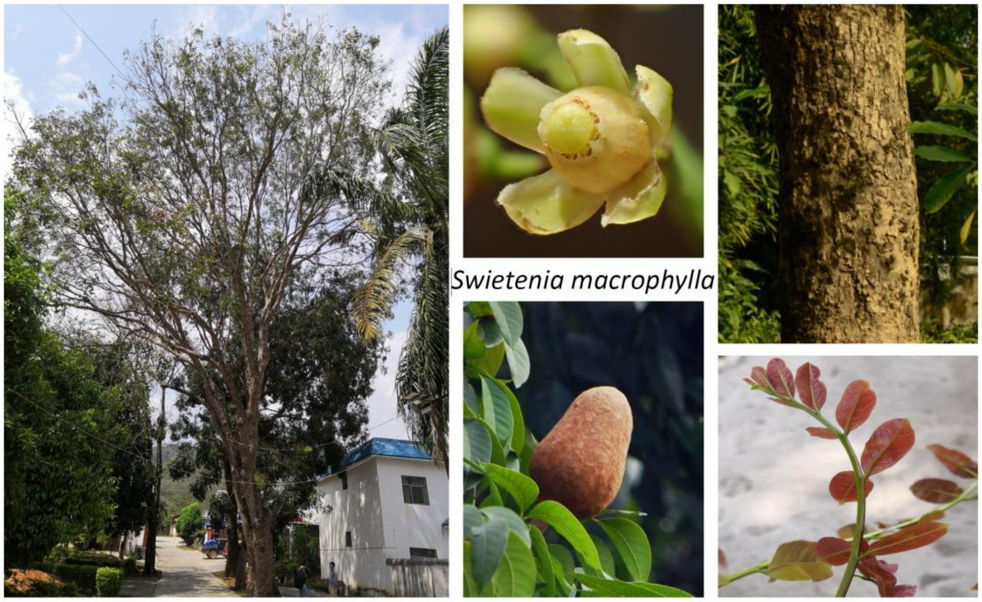

Supplemental Figure 1. Morphological features of *Swietenia macrophylla*

Photos of flower and fruit are obtained from

https://www.inaturalist.org/observations/63391559

https://www.inaturalist.org/observations/24971985

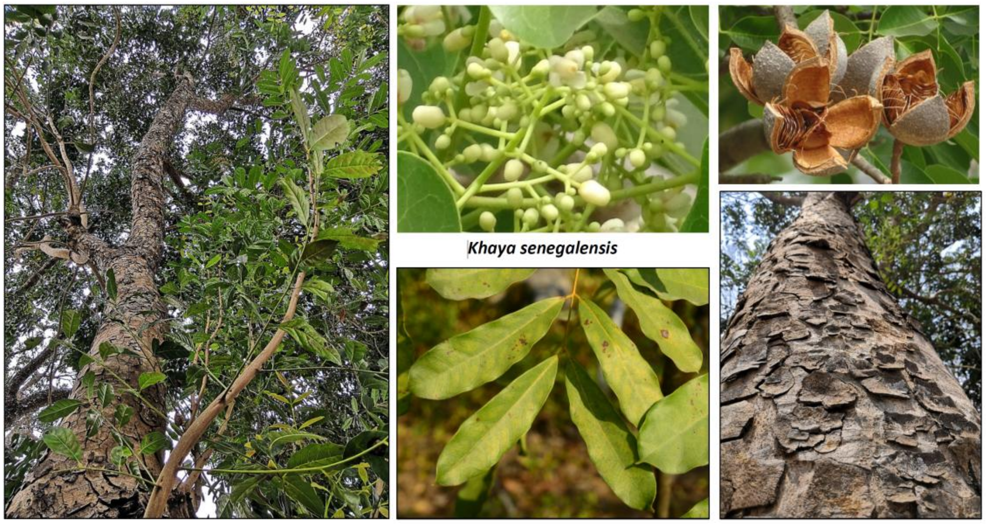

Supplemental Figure 2. Morphological features of *Khaya senegalensis*
Photos of flower and fruit are obtained from
https://www.inaturalist.org/observations/69098325

Table S1 DNA Sequencing statistics of two species

Species	Library construction strategy	Raw reads pair number	Raw base pair number	Raw reads number	Raw base number (bp)	Raw Read length (bp)
Khaya senegalensis	10X	570,609,969	85,591,495,350	1,141,219,938	171,182,990,700	150
	Hi-C	759,897,146	75,989,714,600	1,519,794,292	151,979,429,200	100
Swietenia macrophylla	10X	641,510,428	96,226,564,200	1,283,020,856	192,453,128,400	150
	Hi-C	741,812,693	74,181,269,300	1,483,625,386	148,362,538,600	100

Table S2 RNA sequencing statistics of two species

Species	Tissue	Raw reads pair number	Raw base number	Clean reads pair number	Clean base number	Raw Read length (bp)
Khaya senegalensis	leaf	64,227,120	19,268,136,000	64,143,553	18,425,555,352	150
	xylem1	74,325,184	14,865,036,800	74,150,152	14,082,827,953	100
	xylem2	78,350,807	15,670,161,400	78,223,106	14,857,280,545	100
	xylem3	71,635,114	14,327,022,800	71,527,896	13,585,686,696	100
	phloem1	89,716,011	17,943,202,200	89,540,646	17,006,980,561	100
	phloem2	72,111,286	14,422,257,200	71,972,663	13,670,541,492	100
	phloem3	68,148,636	13,629,727,200	67,934,285	12,901,554,217	100
Swietenia macrophylla	leaf	56,982,281	17,094,684,300	87,583,797	16,629,064,964	150
	xylem	77,568,544	15,513,708,800	77,370,930	14,694,516,379	100
	phloem	76,864,957	15,372,991,400	76,676,834	14,563,059,157	100

Table S3 k-mer analysis of *Khaya senegalensis*, *Swietenia macrophylla*

Species	kmer	raw_peak	genome size	heterozygous ratio	repeat ratio
Khaya senegalensis	21	90	406,501,000	0.73%	42.60%
Swietenia macrophylla	21	123	274,489,000	1.00%	20.14%

Table S4 The genome assemly results of *Khaya senegalensis*, *Swietenia macrophylla*

		Khaya senegalensis				*Swietenia macrophylla*			
		**Contig**	**Scaffold**	**Hi-C Contig**	**Hi-C Scaffold**	**Contig**	**Scaffold**	**Hi-C contig**	**Hi-C scaffold**
**Length (bp)**	N90	2,705	3,549	2,668	3,429	8,220	9,569	8,149	9,375
	N80	7,782	10,342	7,599	9,898	31,167	1,702,943	29,661	6,790,372
	N70	15,985	374,000	15,000	125,000	57,557	3,044,426	54,962	7,228,676
	N60	29,451	1,328,405	25,728	6,785,394	84,179	5,136,928	81,638	7,881,878
	**N50**	**45,659**	**2,533,462**	**40,489**	**7,098,500**	**110,842**	**5,758,776**	**108,891**	**8,511,416**
	N40	64,779	4,201,832	55,523	8,146,634	139,687	7,125,297	137,783	8,986,794
	N30	86,513	5,447,807	75,871	8,792,228	173,247	7,949,705	171,441	9,504,661
	N20	115,035	7,903,176	102,601	9,309,318	216,783	8,746,502	212,398	9,975,239
	N10	163,926	8,986,309	147,164	9,757,406	287,250	9,811,634	280,926	14,894,579
**Number**	N90	14,346	7,798	15,102	7,989	3,808	869	3,903	768
	N80	7,257	1,645	7,885	1,734	2,021	42	2,079	26
	N70	4,212	107	4,722	130	1,388	30	1,422	22
	N60	2,685	55	3,058	26	1,001	23	1,020	18
	N50	1,789	34	2,029	21	720	18	731	14
	N40	1,172	23	1,329	16	503	13	510	11
	N30	731	15	822	12	327	9	331	8
	N20	395	9	445	8	185	6	188	5
	N10	151	4	171	4	75	3	76	2
Maximum length		453,137	11,110,421	377,592	12,119,278	640,644	15,468,293	640,644	15,099,774
Total length		331,727,067	377,763,054	331,726,582	369,939,925	272,898,649	290,208,927	272,898,173	287,673,742
Total number>=100bp		62,615	54,771	63,630	54,893	23,653	19,971	23,858	19,920
Total number>=2000bp		16,772	12,132	17,425	12,062	8,021	5,237	8,116	5,110
Percentage of N content(%)			12.19		10.33		5.96		5.13

Table S5 Busco assessment of genome assembly of scaffold

**BUSCOs**	***Khaya senegalensis***		***Swietenia macrophylla***	
	**NO.**	**P,%**	**NO.**	**P,%**
Complete BUSCOs	**1,323**	**96.2**	**1335**	**97.0**
Complete single copy	1,022	74.3	1080	78.5
Complete duplicated	301	21.9	255	18.5
Fragmented	21	1.5	12	0.9
Missing	31	2.3	28	2.1
Total	1375	100	1375	100

Table S6 The repaeat annotation

Repeat Type	*Khaya senegalensis*		*Swietenia macrophylla*	
	**% in genome**	**Length (bp)**	**%in genome**	**Length (bp)**
SINE	0.03	147,284	0.08	263,198
LINE	0.54	2,083,942	0.48	1,416,342
LTR Copia	9.04	34,812,994	6.24	18,385,381
LTR Gypsy	4.87	18,776,751	5.19	15,295,668
LTR total	16.13	62,173,775	13.15	38,788,013
DNA	4.77	18,398,988	2.93	8,654,436
Satellite	0.03	149,272	0.06	177,417
Simple repeat	0.86	3,317,974	0.12	377,442
Unknown	9.56	36,852,027	6.36	18,761,431
Total	29.5	113,634,213	21.83	64,340,915

Table S7 Model plant or related species in gene annotation

Organism	Annotation version	Downloaded database	Paper URL
Arabidopsis thaliana	TAIR10	https://www.ncbi.nlm.nih.gov/datasets/taxonomy/3702/	—
Anacardium occidentale	v0.9	https://www.ncbi.nlm.nih.gov/datasets/taxonomy/171929/	https://www.nature.com/articles/s41598-022-22600-7
Citrus sinensis	v1.1	https://www.ncbi.nlm.nih.gov/datasets/taxonomy/2711/	https://academic.oup.com/hr/article/10/1/uhac247/6794931
Citrus maxima	—	https://ngdc.cncb.ac.cn/bioproject/browse/insdc/PRJNA796621	https://academic.oup.com/hr/article/doi/10.1093/hr/uhac175/6655783
Dimocarpus longan	—	https://ngdc.cncb.ac.cn/gwh/Assembly/21447/show	https://academic.oup.com/hr/article/doi/10.1093/hr/uhac021/6532241
Eucalyptus grandis	v1.0	https://jgi.doe.gov/	—

Table S8 The result and busco assessment of gene annotation

Table S8-1 The result of gene annotation

	Khaya senegalensis	***Swietenia macrophylla***
Protein-coding gene number	32,914	34,129
Mean gene length (bp)	3,068.00	3,052.92
Mean cds length (bp)	1,217.50	1,204.82
Mean exons per gene	5.29	5.58
Mean exon length (bp)	230.06	215.60
Mean intron length (bp)	431.15	402.79

Table S8-2 Busco assessment of gene annotation

**BUSCOs**	***Khaya senegalensis***		***Swietenia macrophylla***	
	**NO.**	**P,%**	**NO.**	**P,%**
Complete BUSCOs	**1,268**	**92.2**	**1,284**	**93.4**
Complete single copy	1,034	75.2	1,055	76.7
Complete duplicated	234	17	229	16.7
Fragmented	46	3.3	29	2.1
Missing	61	4.5	62	4.5
Total	1375	100	1375	100

Table S9 The functional annotation

	Swietenia macrophylla		*Khaya senegalensis*	
	**Number of genes**	**Percentage (%)**	**Number of genes**	**Percentage (%)**
Nr-Annotated	33,193	97.26	31,386	98.36
Swissprot-Annotated	27,014	79.15	26,004	81.50
KEGG-Annotated	24,807	72.69	23,855	74.76
COG-Annotated	12,664	37.11	12,568	39.39
TrEMBL-Annotated	32,924	96.47	31,309	98.12
Interpro-Annotated	26,063	76.37	25,304	79.30
Overall	33,255	97.44	31,426	98.49
Unannotated	874	2.56	482	1.51

Table S10 The Annotation of non-coding RNA genes in the genomes of *Swietenia macrophylla*, *Khaya senegalensis*

Species		miRNA	tRNA	rRNA					snRNA			
				Total rRNA	18S	28S	5.8S	5S	Total snRNA	CD-box	HACA-box	splicing
Khaya senegalensis	Copy(w)	189	844	630	390	154	24	62	381	225	64	92
	Average length (bp)	123	76	186	238	98	120	97	113	96	125	147
	Total length (bp)	23,156	63,790	116,988	92,966	15,154	2,868	6,000	43,138	21,681	7,970	13,487
	% of genome	0.61%	1.69%	3.10%	2.46%	0.40%	0.08%	0.16%	1.14%	0.57%	0.21%	0.36%
Swietenia macrophylla	Copy (w)	187	648	249	54	119	6	70	324	182	50	92
	Average length (bp)	123	75	142	293	96	118	105	116	95	127	152
	Total length (bp)	22,946	48,817	35,343	15,808	11,456	708	7,371	37,570	17,206	6,348	14,016
	% of genome	0.79%	1.68%	1.22%	0.54%	0.39%	0.02%	0.25%	1.29%	0.59%	0.22%	0.48%

Table S11 DNA reads mapped to the genome

**Species**	**mapped reads rate**	**properly mapped paired reads**
Khaya senegalensis	97.68%	82.72%
Swietenia macrophylla	97.43%	90.55%
